# Myosin 1b flattens and prunes branched actin filaments

**DOI:** 10.1242/jcs.247403

**Published:** 2020-09-24

**Authors:** Julien Pernier, Antoine Morchain, Valentina Caorsi, Aurélie Bertin, Hugo Bousquet, Patricia Bassereau, Evelyne Coudrier

**Affiliations:** 1 Laboratoire Physico-Chimie Curie, Institut Curie, PSL Research University, CNRS UMR168, 75005 Paris, France; 2 Sorbonne Université, 75005 Paris, France; 3 Laboratory Cell Biology and Cancer, Institut Curie, PSL Research University, C.N.R.S. UMR 144, 26 rue d'Ulm, Paris, France; 4 Abbelight, 191 Avenue Aristide Briand, 94230 Cachan, France

**Keywords:** Myosin 1b, Myosin 2, Branched actin, Arp2/3, Gliding assay, TIRF microscopy, Actin architecture

## Abstract

Motile and morphological cellular processes require a spatially and temporally coordinated branched actin network that is controlled by the activity of various regulatory proteins, including the Arp2/3 complex, profilin, cofilin and tropomyosin. We have previously reported that myosin 1b regulates the density of the actin network in the growth cone. Here, by performing *in vitro* F-actin gliding assays and total internal reflection fluorescence (TIRF) microscopy, we show that this molecular motor flattens (reduces the branch angle) in the Arp2/3-dependent actin branches, resulting in them breaking, and reduces the probability of new branches forming. This experiment reveals that myosin 1b can produce force sufficient enough to break up the Arp2/3-mediated actin junction. Together with the former *in vivo* studies, this work emphasizes the essential role played by myosins in the architecture and dynamics of actin networks in different cellular regions.

This article has an associated First Person interview with the first author of the paper.

## Abstract (precis)

**Summary:** *In vitro* F-actin gliding assays and TIRF microscopy reveal that myosin flattens the Arp2/3-dependent actin branches up to the point of them breaking, and reduces the probability that new branches are formed.

## INTRODUCTION

Many motile and morphological processes involve spatially and temporally coordinated branching of filamentous actin (F-actin) networks. Polymerization and assembly of branched networks are controlled by the activity of regulatory proteins, including the Arp2/3 complex (Blanchoin et al., 2014), profilin (Pernier et al., 2016), cofilin (Chan et al., 2009) and tropomyosin (Blanchoin et al., 2001). In addition, several myosin motors have been reported to control actin architecture, including myosins that are bound to cell membranes, such as myosin 1 (see below), myosin 6 (Loubéry et al., 2012; Reymann et al., 2012; Ripoll et al., 2018) and myosin 10 (Nagy et al., 2008), and those associated with F-actin only, such as myosin II (Haviv et al., 2008; Mendes Pinto et al., 2012; Reymann et al., 2012).

**Fig. 1.**
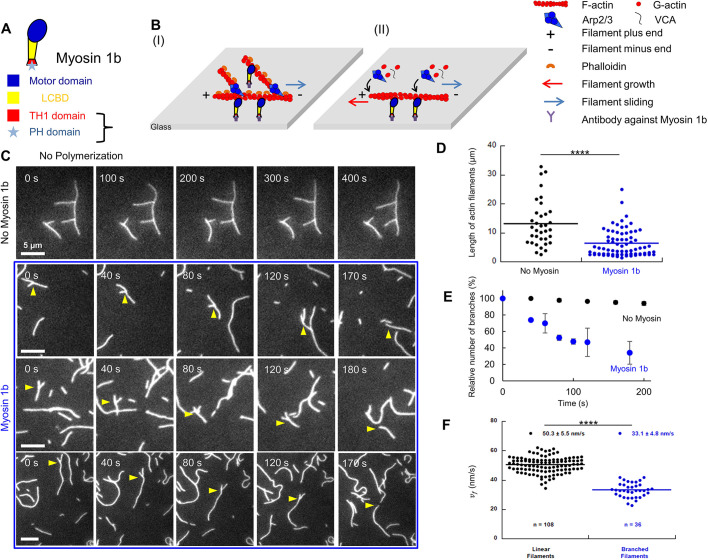
**Sliding on Myo1b reduces branching in stabilized F-actin.** (A) Myo1b domain organization. (B) Sketch illustrating the gliding assays for phalloidin-stabilized branched (I), and polymerizing and branching (II) F-actin sliding on Myo1b anchored to a coverslip. (C) Representative time-lapse images of phalloidin-stabilized branched F-actin sliding or not on Myo1b (from Movie 1). Yellow arrowheads point to a single branch. Scale bars: 5 µm. (D) Distribution of F-actin (mother and daughter) lengths at *t*=160 s in one field corresponding to Movie 1 (with no myosin and Myo1b). A two-tailed *t*-test (*P*=2.08×10^−5^) shows a significant difference between the conditions. (E) Mean±s.e.m. relative number of branches along F-actin filaments (see Materials and Methods) normalized by N_0_, the branch number at *t*=0, over time in the absence of Myo1b (*N*_0_=60, from two movies) or when sliding on Myo1b (N_0_=38, two movies). (F) Dot plot of sliding velocities *v_f_* of stabilized F-actin in the presence of Arp2/3 showing or not showing branches, analyzed from the same movies (five movies). Number of analyzed filaments and mean±s.e.m. are indicated. A two-tailed *t*-test (*P*=2.89×10^−27^) shows a significant difference between data sets. ****P<0.0001.

Myosin 1 proteins are single-headed motors containing three domains: a N-terminal motor domain that coordinates ATP hydrolysis, actin binding and force generation, a light chain binding domain (LCBD) that binds calmodulin, and a tail region with a highly basic C-terminal tail homology 1 (TH1) (McIntosh and Ostap, 2016) (Fig. 1A). In contrast to non-muscle myosin II, a pleckstrin homology (PH) motif in the TH1 domain targets myosin 1 proteins to phosphoinositides in membranes (McConnell and Tyska, 2010) (Fig. 1A). In *Saccharomyces cerevisiae*, myosin 1 (Myo5) induces actin polymerization in a manner that is dependent on the Arp2/3 complex (Sirotkin et al., 2005). This myosin 1 exhibits a SH3 domain at its C-terminus, which binds the WASP yeast homolog, followed by an acidic motif binding Arp2/3 (Evangelista et al., 2000). Studies in *Xenopus* oocytes suggest that myosin 1c and myosin 1e control cortical granule exocytosis and the subsequent compensatory endocytosis by directly regulating actin polymerization (Schietroma et al., 2007; Sokac et al., 2006; Yu and Bement, 2007). Several members of this myosin family in mammals are also involved in organization of actin networks. Myosin 1c stabilizes actin around the Golgi complex (Capmany et al., 2019). Myosin 1b (Myo1b) regulates neuronal polarization and axon elongation by limiting the extension of branched actin networks and favoring actin bundles in growth cones (Iuliano et al., 2018). However, the mammalian myosin 1 proteins lack a SH3 domain or an acidic motif that could interact with Arp2/3. We have recently reported that single F-actins depolymerize when they slide on immobilized Myo1b (Pernier et al., 2019), but how mammalian myosin 1 acts to reorganize branched actin network architecture remains to be explored.

Here, we used *in vitro* F-actin gliding assays and total internal reflection fluorescence (TIRF) microscopy to study how (i) the organization and (ii) the dynamics of both stabilized and polymerizing branched F-actin are modified when sliding on full-length Myo1b immobilized on a substrate (Fig. 1B). We compared the effect of Myo1b in these experimental conditions with that of another myosin, the muscle myosin II (herein referred to as Myo2), which moves linear F-actin five-fold faster (Pernier et al., 2019), keeping in mind that these experimental conditions mimic the physiological topology reported for Myo1b but not for Myo2.

## RESULTS AND DISCUSSION

### The branch density of stabilized F-actin decreases when sliding on Myo1b

**Fig. 2.**
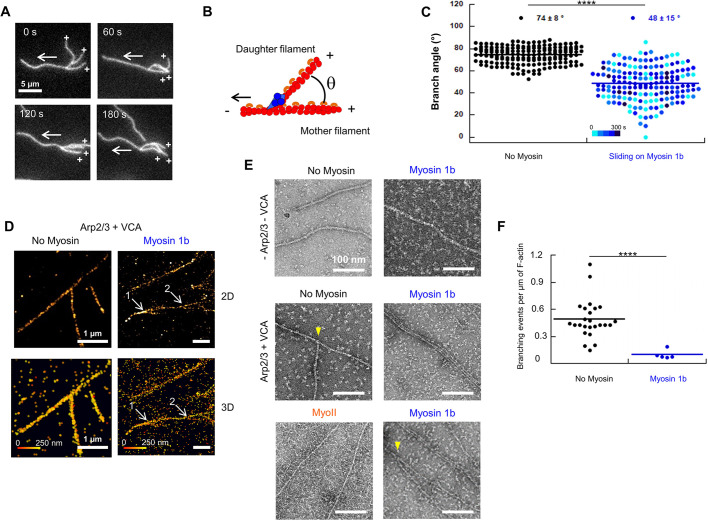
**Myo1b reduces stabilized F-actin angles in branched structures.** (A) Time-lapse images of stabilized branched F-actin, sliding on Myo1b, showing a change of the angle between mother and daughter filaments (Movie 3). Crosses indicate barbed ends and white arrows the sliding direction. Scale bar: 5 µm. (B) Representation of the θ angle between mother and daughter filaments. (C) Dot plot of θ angle for filaments without myosin (12 filaments, 168 images) or sliding on Myo1b (19 filaments, 168 images). The blue color scale indicates the time since acquisition started. A two-tailed *t*-test (*P*=6.16×10^−51^) shows a significant difference between the conditions. (D) STORM images of stabilized branched F-actin in 2D (top) or 3D (bottom) without or with Myo1b. These images correspond to the squares in Fig. S1A. Color code indicates the height (*z*). Without Myo1b, branch and mother filaments are in the same plane and attached. On Myo1b, branches #1 and #2 exhibit a θ angle that is much lower than 70°, but are physically connected to the mother filament. Note that branch #2 is in a lower plane. (E) Electron microcopy images of stabilized F-actin, branched or not, recorded after gliding for 10 min or not on Myo1b or Myo2 and after negative staining. These images correspond to the squares in Fig. S1B. Scale bars: 100 nm. Yellow arrowheads point to a single branch. (F) Dot plot representing the number of branches per µm of F-actin, in the absence of Myo1b (*n*=25) or when sliding on Myo1b (*n*=5), quantified from electron microscopy images. A two-tailed *t*-test (*P*=9.78×10^−9^) shows a significant difference. ****P<0.0001.

We first polymerized F-actin in solution in the presence of the constitutively active C-terminal domain of N-WASP (VCA) and the Arp2/3 complex (see Materials and Methods) (Pernier et al., 2016), to generate branched filaments and stabilized them with phalloidin. We analyzed the architecture of the filaments, and their movement when sliding on Myo1b bound to a glass substrate at a density of a 8000/µm^2^ (Pernier et al., 2019) (Fig. 1C; Movie 1). Without Myo1b and with the same surface treatment, the branches were stable for at least up to 10 min (Movie 1; Fig. 1C,E). In contrast, when filaments slide on motors, we observe large variations of the orientation of the branches relative to the mother filaments, that are already visible 1 or 2 min after gliding (e.g. Movie 1, Figs 1C and 2C) and detachment of branches from the mother at the level of the branch attachment (Movie 1, Fig. 1C, last frames). Quantification of the length distribution of the F-actin filaments after 160 s shows an important and significant (*P*=2.08×10^−5^) increase in the proportion of short filaments when gliding on Myo1b (Fig. 1D), while the total length of filaments (including mother, daughter and single filaments) is conserved throughout the period of imaging (at 457 and 459 µm, respectively; *n*=34 at *t*=0 s, *n*=35 at *t*=160 s), which is similar to what is found for the control (448 and 459 µm). On average, the filament length is 13.1 µm without Myo1b and 6.2 µm in its presence (Fig. 1D). Since filaments are stabilized by treatment with phalloidin, the reduction of their length when sliding on Myo1b cannot be due to its actin depolymerase activity (Pernier et al., 2019). It is also not due to a severing activity of Myo1b; we observed that 100% of the breaks occur at the base of the shorter (daughter) filament within the TIRF resolution (1 pixel=160 nm). In contrast, severing would occur everywhere along mother and daughter filaments. Moreover, we did not detect severing on stabilized linear filaments (Pernier et al., 2019). Thus, our observations rather suggest that sliding on Myo1b induces detachment of branches.

As a comparison, we used Myo2, which leads to a five-fold faster linear F-actin sliding (Pernier et al., 2019). At the same surface density, no branches on filaments gliding on Myo2 were observed, but we could see F-actin fragments; this is likely due to a fast detachment occurring before image acquisition started, that is at 1 min after injection of branched filaments in the chamber (Movie 2). Since we aimed at characterizing the debranching process mediated by myosins, given the time scale of our experiments, we focused on the analysis of Myo1b for the rest of the study.

We next quantified the relative variation of the number of branches along F-actin over time. The branch number decreases by five-fold within 3 min on Myo1b (Fig. 1E). The presence of branches reduced the gliding velocity, from 50.3±5.5 nm/s (mean±s.e.m.) for filaments without branches, as we previously reported (Pernier et al., 2019; Yamada et al., 2014), down to 33.1±4.8 nm/s for branched F-actin (Fig. 1F).

### Sliding on Myo1b flattens the branches on the actin filaments

Because we observed variations in the orientation the branches (Figs 1C and 2A; Movies 1 and 3), we measured the θ angle between mother and daughter filaments (Fig. 2B) on a large collection of snapshots, without or with Myo1b. On average, θ=74±8° (mean±s.e.m.) in control conditions, in agreement with previous reports (Mullins et al., 1998; Svitkina and Borisy, 1999) (Fig. 2C). In contrast, with Myo1b, θ decreased to 48±15°, with a large spreading of values down to almost zero (Fig. 2C). This large spreading is maintained over 5 min of observation (Fig. 2C, blue scale), reflecting a large flexibility of the branched filaments architecture when sliding on Myo1b. In many instances the branch flattens on the mother (Movies 1 and 3) and the angle decays to zero; this means that the branch is ‘falling’ back towards the main filament axis in the direction opposite to the movement. Using 3D stochastic optical reconstruction microscopy (STORM), we confirmed that even when θ is close to zero, the branch remains attached to the mother filament, and is not juxtaposed to it (Fig. 2D; Fig. S1A).

We next analyzed the architecture of the actin networks with electron microscopy (Fig. 2E; Fig. S1B). Fig. 2E shows a representative image of a branched filament without Myo1b with θ=67°, of a bundle of three parallel filaments and of a branch with a reduced angle with Myo1b. On average, there were 0.48 branches/µm without Myo1b (*n*=25 fields of 2.5×1.7 µm^2^), and 0.1 branches/µm with Myo1b (*n*=5) (Fig. 2F). In agreement with our time-lapse observations (Movie 2), we observed neither branches nor bundles with Myo2 (Fig. 2E).

Taken together, these observations highlight that Myo1b has the capability to modify the architecture of branched stabilized actin networks, to reduce the branching angle down to zero in the direction opposite to the movement, and even to break Arp2/3-mediated branches.

### Sliding on Myo1b reduces branching dynamics and induces debranching

**Fig. 3.**
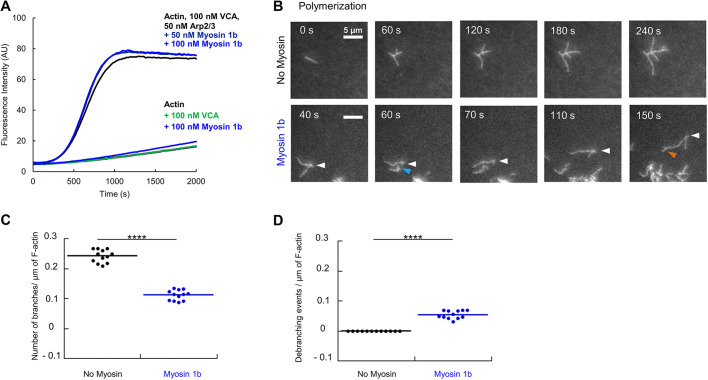
**Sliding on Myo1b decreases branching of polymerizing actin filaments.** (A) Effect of Myo1b on branched F-actin polymerization as determined using a pyrene assay. (B) Time-lapse images of polymerizing and branching F-actin in the absence of Myo1b or when sliding on Myo1b with 2 mM ATP (Movie 4). Scale bars: 5 µm. White, cyan and orange arrows point to a mother filament with debranching and branching events, respectively. (C) Numbers of branches per µm of F-actin, without myosin or when sliding on Myo1b. Data are obtained from the total number of branches detected at different times (Fig. S2A) normalized by the corresponding total length of F-actin filaments (Fig. S2C) (three movies). A two-tailed *t*-test (*P*=1.47×10^−13^) shows a significant difference between the conditions. (D) Numbers of debranching events per µm of F-actin, without or with Myo1b, obtained from the same normalization as in C, using data shown in Fig. S2B,C. A two-tailed *t*-test (*P*=2.61×10^−8^) shows a significant difference between the conditions. ****P<0.0001.

We next investigated the effect of Myo1b on dynamical filaments that polymerize and branch, using bulk pyrene-actin fluorescence or TIRF microscopy assays. As previously reported for linear F-actin, Myo1b in solution does not change the polymerization of branched F-actin (Fig. 3A) (Pernier et al., 2019). Using the TIRF assay, we observed the growth of long and straight branches along the growing mother filament in the absence of myosin, as expected (Fig. 3B; Movie 4). In contrast, when sliding on Myo1b, the growing filaments appeared very distorted. As previously reported in the absence of Arp2/3 (Pernier et al., 2019), Myo1b also reduced the actin polymerization rate in these conditions. It decreased from 7.37±0.57 subunits (su)/s (mean±s.e.m.; *n*=60) without myosin (Pernier et al., 2019) down to 5.32±0.66 su/s (*N*=25) with Myo1b. Branches also formed over time when sliding on Myo1b but they frequently detached and ‘ran away’ (Fig. 3B; Movie 4, Fig. S2A). We normalized the branch number measured at different times by the corresponding total length of F-actin filaments (Fig. S2C). The number of branches formed per µm was time-independent but was reduced by a factor of 2 (0.11±0.02 µm^−1^; mean±s.e.m.) when sliding on Myo1b as compared to the control (0.24±0.02 µm^−1^) (Fig. 3C). The cumulative number of debranching events increased over time when sliding on Myo1b while no events occured in control conditions (Fig. S2B). When normalized by the total length of F-actin filaments (Fig. 3D), the density of debranching events was 0.05±0.01 µm^−1^. The observed normalized branch number (Fig. 3C) corresponds to the difference between the normalized branch numbers effectively formed (Fig. S2D) and those that detach (Fig. 3D). Results in Fig. 3C,D show that the effective number of newly formed branches is equal to 0.16±0.02 µm^−1^, lower than in the control (Fig. S2D). This indicates that sliding of F-actin on Myo1b, besides reducing polymerization rate, induces debranching and also reduces the probability of generating new branches.

**Fig. 4.**
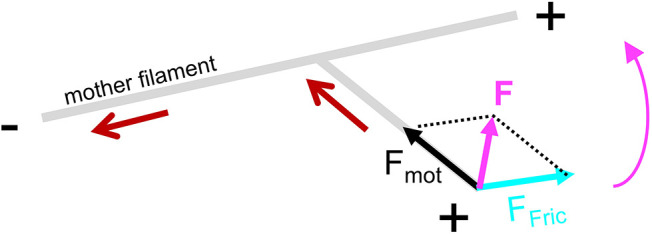
**Scheme of the forces exerted on a daughter filament.** Mother filament sliding (red arrow) induces a friction force (*F_fric_*) on the daughter filament. *F_fric_* combined with the force generated by the motors on the branch (*F_mot_*) results in a total force **F** at the extremity of the filament, and thus in a torque leading to the reduction of the angle between both filaments (magenta arrow).

It is generally accepted that both of the actin network types, branched and parallel network, which depend on Arp2/3 complex and formins for their formation, respectively, compete for the same G-actin pool (Chesarone and Goode, 2009). More recently, it has been shown that accessory proteins, such as profilin and capping protein, can interfere and favor the polymerization of one or the other network type (Rotty et al., 2015; Suarez et al., 2015). In this report, we show that beside the accessory proteins, Myo1b and Myo2 can also remodel the architecture of branched actin networks when attached to a substrate. This is not the case when myosins are free in solution and cannot exert forces. Bieling and colleagues (Bieling et al., 2016) suggested that compressive forces on a branched actin gel alter the internal architecture of the network, increasing its density. Recent work using microfluidics (Pandit et al., 2020) confirms that Arp2/3-dependent branches can break when forces in the pN range are applied. In our experiments on stabilized and dynamic F-actin, the mother filament and the branches are propelled by myosins in divergent directions owing to the geometry of the Arp2/3-generated branch, which induces friction forces on the filaments (Fig. 4). This explains the reduction of sliding velocity in the presence of branches. Moreover, a combination of the friction force (*F_fric_*) on the branch due to mother filament sliding and the force produced by motors on this branch (*F_mot_*) results in a force **F** at the filament extremity, and thus a torque that reduces the θ angle (Fig. 4). Consequently, the Arp2/3 junction is submitted to a stress and, depending on the stiffness of the Arp2/3–F-actin junction, the branch might break either at the junction or next to it.

We have also shown that the probability of forming new branches on the side of a mother filament is reduced when sliding on myosins. Tropomyosin (Blanchoin et al., 2001), cofilin (Chan et al., 2009) and coronin (Cai et al., 2008) inhibit the polymerization of Arp2/3-dependent actin branches by competing with the Arp2/3 complex for their binding site along F-actin. Similarly, Myo1b, which also binds along F-actin (Mentes et al., 2018), might compete with Arp2/3 for the same binding site.

In cells, Myo1b is bound to membranes. The Ostap group and us (Pernier et al., 2019; Pyrpassopoulos et al., 2012) have reported that even when Myo1b is bound to fluid lipid bilayers, it can propel F-actin due to the high membrane viscosity (Pernier et al., 2019). Thus, it is very likely that Myo1b also mediates debranching and reorganizes the architecture of branched actin networks at the plasma membrane. This is supported by our *in vivo* observations that Myo1b is required for the formation of filopodia induced by EphB2 receptor stimulation (Prospéri et al., 2015) and that it controls the extension of branched actin networks in growth cones (Iuliano et al., 2018). By debranching actin networks near to the plasma membrane, Myo1b may favor the formation of linear and parallel filaments in growth cones and thus facilitate filopodia initiation upon EphB2 receptor stimulation. Finally, we showed that Myo1b can pull membrane tubules along actin bundles (Yamada et al., 2014), which is reminiscent of the Myo1b-dependent tubule elongation at the trans-Golgi network (Almeida et al., 2011). An additional role for Myo1b in this cellular region could be to debranch the Arp2/3 network to form actin structures required for it to pull tubules. Other myosin 1 proteins have been reported to influence actin organization *in vivo* (Capmany et al., 2019; Schietroma et al., 2007; Sokac et al., 2006; Yu and Bement, 2007); however, their motor properties and mechanosensitivity differ from Myo1b (McIntosh and Ostap, 2016), thus they might act differently on actin branches. Myo2 also inhibits the formation of branched actin structures. It is important to stress that this motor is not membrane-bound but associated with contractile fibers. Whether or not Myo2 *in vivo* contributes to formation of contractile fibers by debranching Arp2/3 actin network remains to be studied.

This work, together with former *in vivo* studies, reveals the essential role played by Myo1b in the architecture and in the dynamics of actin networks at the plasma membrane and on cellular compartments. Considering its motor activity produces F-actin motion, we can envisage that Myo1b is also involved in general actin movements and fluxes at larger scales.

## MATERIALS AND METHODS

### Protein purification

Actin was purified from rabbit muscle and isolated in monomeric form in G buffer (5 mM Tris-HCl pH 7.8, 0.1 mM CaCl_2_, 0.2 mM ATP, 1 mM DTT and 0.01% NaN_3_). Actin was labeled with Alexa Fluor 594 succimidyl ester-NHS (Ciobanasu et al., 2014). Myosin II was purified from rabbit muscle as previously described (Pollard, 1982). The Arp2/3 complex was purified from bovine brain (Egile et al., 1999).

Expression and purification of Myosin 1b was undertaken as follows. FLAG–Myo1b (Yamada et al., 2014) was expressed in HEK293-Flp-In cells (Thermo Fisher Scientific) that were regularly tested for contamination, cultured in Dulbecco's modified Eagle's medium (DMEM) supplemented with 10% fetal bovine serum and 0.18 mg ml^−1^ hygromycine in a spinner flask at 37°C under 5% CO_2_, and collected by centrifugation (1000 ***g***, 10 min, 4°C) to obtain a 4–5 g of cell pellet as described by Yamada et al. (2014). The pellet was lysed in FLAG Trap binding buffer [30 mM HEPES pH 7.5, 100 mM KCl, 1 mM MgCl_2_, 1 mM EGTA, 1 mM ATP, 1 mM DTT, 0.1% protease inhibitor cocktail (PIC) and 1% Triton X-100] for 30 min at 4°C and centrifuged at 3400 ***g*** for 10 min at 4°C. The collected supernatant was then ultracentrifuged in a Beckman 70.1 Ti rotor (250,000 ***g***, 60 min, 4°C). The solution between the pellet and floating lipid layer was incubated with 150 µl of anti-FLAG beads for 2 h at 4°C. The beads were collected by centrifugation (1000 ***g***, 5 min, 4°C). After a washing step, FLAG–Myo1b was then eluted by incubating with 0.24 mg ml^−1^ of 3× FLAG peptide in 300 µl elution buffer (binding buffer without Triton X-100 supplemented with 0.1% methylcellulose) for 3 h at 4°C. After removal of the beads by centrifugation (1000 ***g***, 3 min, 4°C), the protein solution was dialyzed against elution buffer overnight at 4°C to remove the 3× FLAG peptide. Myo1b was fluorescently labeled using Alexa Fluor 488 5-SDP ester (Yamada et al., 2014). Inactivated Myo1b was removed by ultracentrifugation in a Beckman TLA-100.3 rotor (90,000 rpm, 20 min, 4°C) with 10 µM F-actin in presence of 2 mM ATP. Inactivated Myo1b was then dissociated from F-actin by incubating the pellet collected after untracentrifugation in elution buffer (30 mM HEPES, pH 7.5, 100 mM KCl, 1 mM MgCl_2_, 1 mM EGTA, 1 mM ATP, 1 mM DTT and 0.1% methylcellulose) supplemented with 1 M NaCl and collected in the supernatant after a second centrifugation in a Beckman TLA-100.3 rotor (90,000 rpm, 20 min, 4°C).

### Actin polymerization assays using a pyrenyl assay

Actin polymerization kinetic experiments were based on measuring the fluorescence change of pyrenyl-labeled G-actin (λ_exc_=365 nm, λ_em_=407 nm). Experiments were carried out on a Safas Xenius spectrofluorimeter (Safas, Monaco). Polymerization assays were performed in F-buffer (5 mM Tris-HCl, pH 7.8, 100 mM KCl, 1 mM MgCl_2_, 0.2 mM EGTA, 0.2 mM ATP, 1 mM DTT and 0.01% NaN_3_) in the presence of 100 nM VCA [from human N-WASP (amino acids 392-505); provided by the Carlier laboratory], 50 nM Arp2/3 and 2 µM actin with 10% labeled with pyrenyl.

### TIRF microscopy assays and data analysis

To measure the kinetics of single filament assembly, coverslips and glass slides were sequentially cleaned by sonication with H_2_O, ethanol, acetone for 10 min, and then 1 M KOH for 20 min and H_2_O for 10 min. Flow chambers were assembled with a coverslip bound to a glass slide with two parallel double-stick tapes. The chamber was incubated with 100 nM anti-Myo1b antibody (Almeida et al., 2011) in G buffer (5 mM Tris-HCl, pH 7.8, 0.1 mM CaCl_2_, 0.2 mM ATP, 1 mM DTT and 0.01% NaN_3_) for 10 min at room temperature. The chamber was rinsed three times with buffer G 0.1% BSA and incubated for 5 min at room temperature. Then the chamber was incubated with 300 nM Alexa Fluor 488-labeled Myo1b in Fluo F buffer (5 mM Tris-HCl pH 7.8, 100 mM KCl, 1 mM MgCl_2_, 0.2 mM EGTA, 0.2 mM or 2 mM ATP, 10 mM DTT, 1 mM DABCO and 0.01% NaN_3_) for 10 min at room temperature. Actin gliding assays were performed in Fluo F buffer, containing ATP maintained at a constant concentration of 2 mM with a regenerating mix (see below), supplemented with 0.3% methylcellulose (Sigma) and with G-actin (1 μM, 10% labeled with Alexa Fluor 594), VCA and Arp2/3 complex or stabilized branched F-actin (stabilized with phalloidin–Alexa Fluor 594) prepared as below. Branched F-actin was polymerized in the presence of 1 µM G-actin, 100 nM VCA and 50 nM of Arp2/3 complex. In the case of stabilized filaments, after 10 min, samples were diluted 20-fold to 50 nM and supplemented with phalloidin. The dynamics of stabilized branched filaments and polymerizing and branching actin filaments was monitored by TIRF microscopy (Eclipse Ti inverted microscope, 100× TIRF objectives, Quantem 512SC camera). The experiments were controlled using the Metamorph software. To maintain a constant concentration of ATP in this assay, an ATP regenerating mix, including 2 mM ATP, 2 mM MgCl_2_, 10 mM creatine phosphate and 3.5 U/ml creatine phosphokinase, which constantly re-phosphorylates ADP into ATP to maintain a constant concentration of free ATP, was added. Actin polymerization was measured as described by (Pernier et al., 2019) and expressed in actin subunits per second (su/s).

The sliding velocities of actin filaments were analyzed by using the ‘Kymo Tool Box’ plugin in Image J software (https://github.com/fabricecordelieres/IJ_KymoToolBox). The accuracy on the displacement of the filaments is of the order of the pixel size (160 nm). The number of branches and the length of the filaments were measured by using the ‘Analyze Skeleton’ plugin in Image J (https://imagej.net/AnalyzeSkeleton) on transformed movies obtained by running an automatic macro freely accessible on http://xfer.curie.fr/get/SItjDQoyu4k/automatization_skeleton.ijm.

Statistical analysis was performed by using Student *t*-test in Microsoft Excel.

### STORM

Stabilized branched F-actin in the absence of myosin or after 10 min of sliding on Myo1b was visualized by incubation with phalloidin–Alexa Fluor 647. Samples were mounted in dSTORM buffer (Abbelight, Paris, France). Then, 3D nanoscopy images were taken using a SAFe360 module (Abbelight) coupled to the camera port of an inverted bright-field Olympus IX71 microscope, equipped with a 100× oil-immersion objective with a high numerical aperture (NA 1.49). This quad-view system (dual-cam sCMOS cameras, Orcaflash v4, Hamamatsu) provided 3D nanoscopy information with high isotropic localization precision (15×15×15 nm, over an axial range of ∼1 mm). Axial information was obtained by super-critical angle fluorescence (SAF) and the point spread function (PSF) deformation with a strong astigmatism (DAISY) (Cabriel et al., 2019). A total of 20,000 frames at 50 ms exposure time were acquired and used for single-molecule detections and to reconstruct a nanoscopy image. Resulting coordinate tables and images were processed and analyzed using SAFe NEO software (Abbelight).

### Electron microscopy

4 µl of Myo1b or Myo2 samples was incubated on glow-discharged and carbon-coated electron microscopy grids (CF-300, EMS). After rinsing with the actin buffer, stabilized branched F-actin polymerized with 4 µM G-actin, 100 nM VCA and 50 nM of Arp2/3 complex was incubated on the grid with 2 mM ATP. The sample was then stained with 4 µl uranyl formate 2%. Images were collected with a Tecnai Spirit transmission electron microscope equipped with a LaB6 emission filament operated at an acceleration voltage of 80 kV (Thermofischer, FEI, Eindhoven, The Netherlands). The microscope is equipped with a Quemesa (Olympus) camera for imaging.

## Supplementary Material

Supplementary information
