# Supplementary material for: Myosin 1b flattens and prunes branched actin filaments
Source: J Cell Sci. 2020 Sep 24;133(18):jcs247403. doi: 10.1242/jcs.247403 (PMC7522023; doi:10.1242/jcs.247403)
Supplement: Supplementary information [file joces-133-247403-s1.pdf]

Supplemental Information

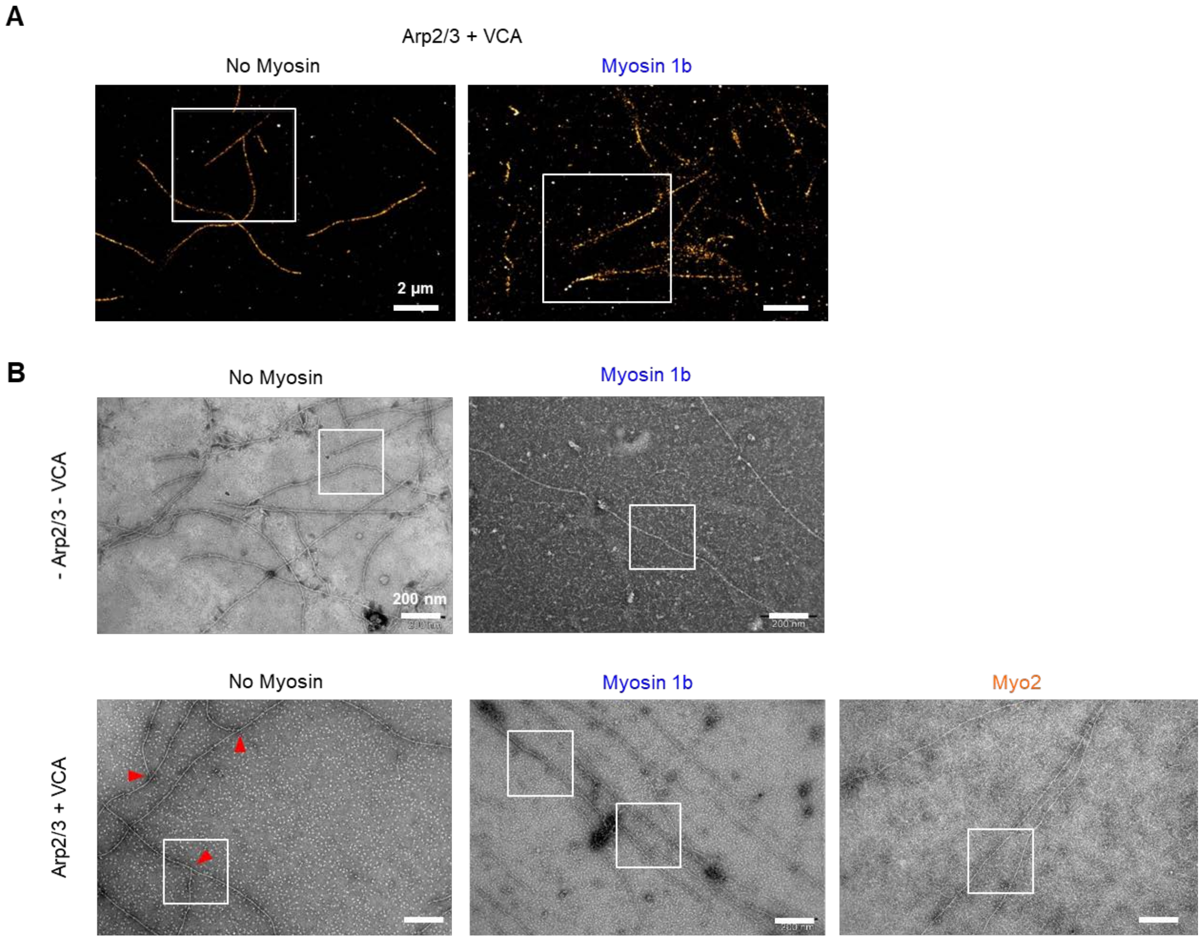

Figure S1: Large fields corresponding to the zooms (white frames) shown: (A) in Fig. 3D with STORM and (B) in Fig. 3E with electron microscopy. In (B), red triangles indicate branched junctions. Scale bars 2 µm (A) and 200 nm (B).

Journal of Cell Science • Supplementary information

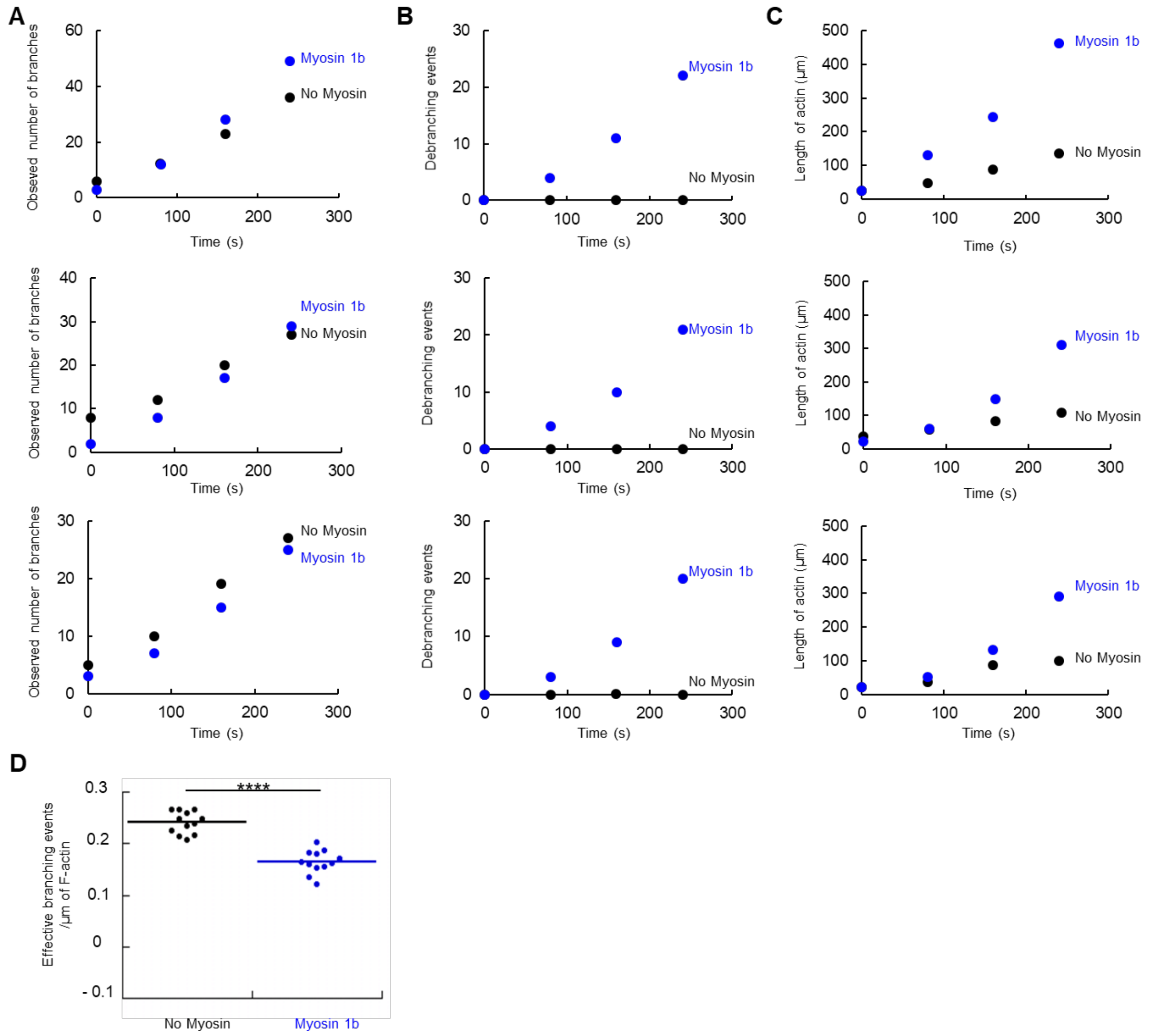

Figure S2: Data corresponding to Fig. 3C and 3D. (A) Observed number of branches, **(B)** total debranching events, **(C)** total length of actin, over time and on three different fields of 80x80 µm^2. **(D)** Effective branching events per µm of F-actin, calculated from the sum of the observed branches number (Fig. 3C) and debranching events (3D), without or with Myo1b. A two-tailed t-test ($p=1.51\times10^{-8}$) shows a significant difference. ****$p < 0.0001$.

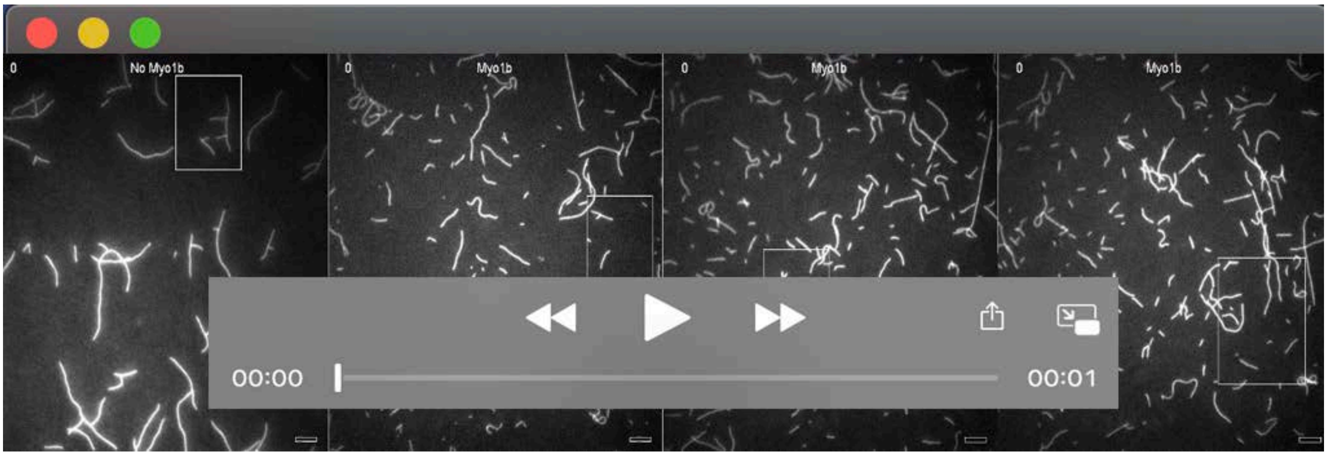

Movie 1: Stabilized branched actin filaments sliding along glass-anchored myosin 1b (corresponding to Fig. 2).

Branched filaments stabilized with phalloidin-Alexa547 in the absence of myosin (left) or sliding on Myo1b at high density (8000 µm $^{-2}$). Scale bar 5 µm. Time in s. Wide field movies corresponding to time-lapse images shown in Fig. 1C.

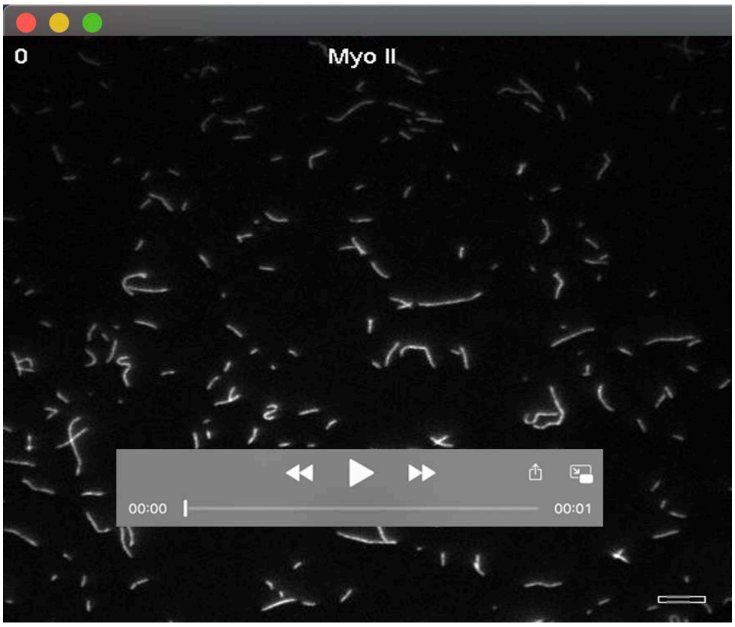

Movie 2: Stabilized branched actin filaments sliding along Myo2.

Branched filaments stabilized with phalloidin-Alexa547 sliding on Myo2. The movie starts 1 min after injection of branched filaments in the chamber. Scale bar, 5 µm. Time in s.

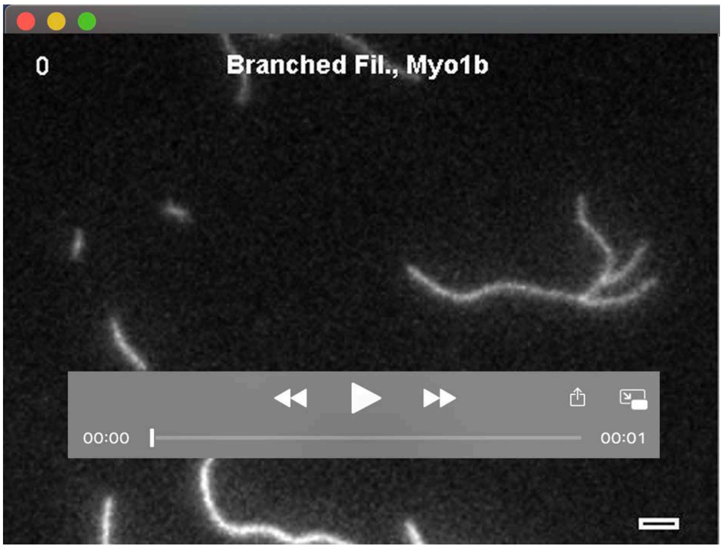

Movie 3: Effect of myosin 1b on organization of branched structures (corresponding to Fig. 2A).

Branched filaments stabilized with phalloidin-Alexa547 sliding on Myo1b. Scale bar, 5 µm. Time in s.

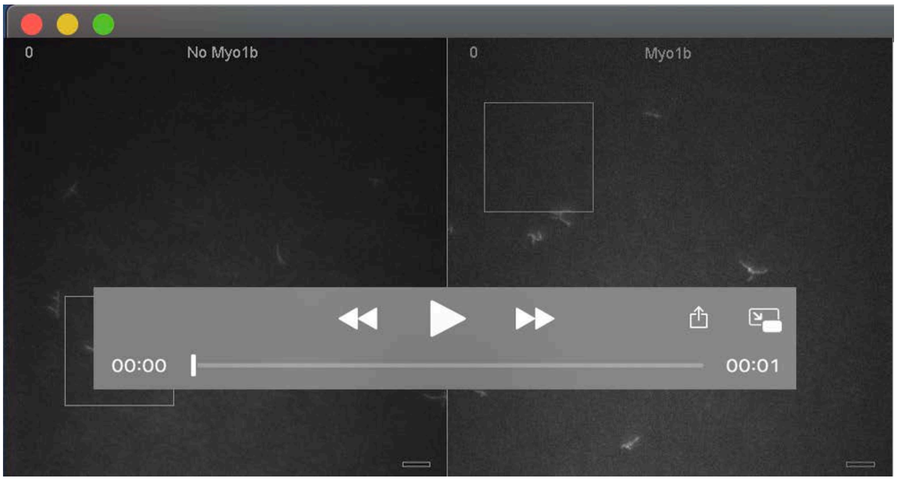

Movie 4: Polymerizing and branching actin filaments sliding along glass-anchored Myo1b (corresponding to Fig. 3).

Polymerized and branched actin filaments in the absence of myosin or sliding on Myo1b at high density (8000 µm^{-2}). Scale bar, 5 µm. Time in s. Wide field movies corresponding to time-lapse images shown in Fig. 3B. White arrow points to a mother filament. The appearance over time of new actin filaments on the right side of the movie likely results from the high affinity of these filaments for the surface coated with Myo1b.
